# Modified Banff Criteria in Assessing SARS-CoV-2-Associated Renal Pathology: An Autopsy Study

**DOI:** 10.7759/cureus.37260

**Published:** 2023-04-07

**Authors:** Hristo Popov, George S Stoyanov, Lilyana Petkova

**Affiliations:** 1 General and Clinical Pathology, Forensic Medicine and Deontology, Medical University of Varna, Varna, BGR; 2 Pathology, Complex Oncology Center, Shumen, BGR

**Keywords:** renal pathology, autopsy, pathology, survival, banff, nephropathology, sars-cov-2

## Abstract

Introduction

SARS-CoV-2 is an epitheliotropic viral agent with epithelial tropism. Although the clinical significance and severity of affection is the most pronounced in the respiratory system, other organs and systems are also infected and, hence affected, such as the central nervous system, gastrointestinal tract, cardiovascular, and urinary systems. Herein, we set out to evaluate the presence and degree of morphological changes within the renal parenchyma and its relation to disease outcome.

Materials and methods

A retrospective non-clinical approach was utilized for the means of the study. All patients with real-time reverse transcriptase-polymerase chain reaction proven infection, subject to an autopsy performed in a period of two calendar years, were included in the study. Kidney tissue histopathology samples were analyzed using a modified Banff criteria system for acute onset and chronic changes. The results were compared for statistical significance with overall patient survival from symptom onset to death. Furthermore, SARS-CoV-2 viral presence was evaluated in renal structures by means of immunohistochemistry.

Results

A total of 40 patients were included in the study. Immunohistochemistry showed viral presence within a myriad of renal structured - endothelial cells, tubular cells, and podocytes. Modified Banff criteria showed significant acute changes within the parenchyma, including endotheliitis, glomerulitis, mesangial matrix expansion, tubulitis, capillaritis, arteritis, thrombosis (including thrombotic microangiopathy in four patients), and hemorrhages. Individual cases also presented with signs of rhabdomyolysis - myoglobulin casts. Signs of chronic injury were also present in most patients. However, when calculated as scores, neither acute nor chronic changes showed a correlation with time from symptom onset to death.

Conclusion

The results of the present study show both viral presence and a myriad of induced changes in the contents of SARS-CoV-2 infection within the renal parenchyma. The lack of correlation with the degree of changes, when compared to survival, is an encouraging fact that the changes are unlikely to play a role in direct tanatogenesis while having the potential to manifest as chronic kidney disease in the future.

## Introduction

SARS-CoV-2, which is the causative agent of the clinical disease entry COVID-19, is an epitheliotropic viral agent with profound tropism to respiratory system epithelia [1]. Although initially thought to be purely a respiratory disease, and while indeed the majority of patients present with respiratory symptoms, which persist as the most severe throughout the disease course and are the most often cause of death in fatal cases, much has been established about the viral tropism to other sites and organs such as the central nervous system, gastrointestinal tract, cardiovascular and urinary system.

One of the key aspects of urinary system disease in the context of COVID-19 is direct damage to the kidneys, at first an overlooked mechanism but one grounded on the basic biological properties of coronaviruses as a whole [1].

Herein, we set out to evaluate the virus-induced properties of SARS-CoV-2 and the model of induced damage in the renal structures in a cohort of virologically proven cases of COVID-19 diseased patients.

## Materials and methods

Study design and cohort

A retrospective non-clinical approach was utilized for the means of the study. All patients with real-time reverse transcriptase-polymerase chain reaction-proven COVID-19, subject to an autopsy performed in the period November 2020-November 2022, were withdrawn from the central pathology repository of St. Marina University Hospital.

Histopathological evaluation

Renal histopathology was evaluated using a modified Banff model (Table 1). The modified scoring system included evaluating endotheliitis, glomerulitis, capillaritis, tubulitis, arteritis, thrombosis, hemorrhages, and interstitial fibrinoid necrosis as acute damage indicators and interstitial inflammation, fibrosis, tubular atrophy, and calcification as indicators for chronic damage. The combined scoring criteria equaled a total score of 13 points, indicating a morphologically normal kidney, with acute damage indicators equaling a total of nine points and chronic damage indicators equaling a total of four points of the total score. Separation of these markers was done with the goal of comparing the acute onset damage within the kidneys that could have occurred only during the course of COVID-19 for the means of statistical analysis, without pre-infection changes altering the statistical score.

**Table 1:** Modified Banff criteria used for the evaluation of renal histopathological changes

*Endotheliitis* *(score)*	*Glomerulitis* *(score) as a percentage of glomeruli affected*	*Mesangial matrix expansion* *(score) as a percentage of glomeruli affected*	*Capillaritis* *(score)*	*Tubulitis* *(score)*	*Arteritis* *(score)*	Thrombosis	Hemorrhages	*Interstitial fibrinoid necrosis* *(score) as a percentage of the total area*	*Interstitial inflammatory infiltration* *(score) as a percentage of the total area*	Fibrosis (score) as a percentage of the total area	Tubular atrophy (score) as a percentage of the total area	Calcification
Absent – 1	Absent – 1	Absent – 1	Absent – 1	Absent – 1	Absent – 1	Absent – 1	Absent – 1	Absent – 1	Absent – 1	Absent – 1	Absent – 1	Absent – 1
Present – 2	<10% – 2	<25% – 2	<5 inflammatory cells – 2	<5 inflammatory cells – 2	<25% luminal stenosis – 2	Present – 2	Present – 2	<25% – 2	<10% – 2	<25% – 2	<25% – 2	Present – 2
	10-25% – 3	26-50% – 3	5-10 inflammatory cells – 3	5-10 inflammatory cells – 3	>25% liminal stenosis – 3			26-50% – 3	10-25% – 3	26-50% – 3	26-50% – 3	
	>26-50% – 4	>51% - 4	>11 inflammatory cells – 4	>11 inflammatory cells – 4	Transmural arteritis with fibrinoid necrosis and leucocytic infiltration – 4			>51% – 4	26-50% – 4	>51% – 4	>51% – 4	

Immunohistochemistry

Immunohistochemistry was performed to evaluate the replication and invasion potential of SARS-CoV-2 within the renal structures with Rabbit polyclonal to SARS-CoV-2 spike glycoprotein antibody (catalog number: ab272504, Abcam, Cambridge, United Kingdom). The antibody was chosen as it reacts to the intracellular and transmembrane domain of the coronavirus spike protein, which, unlike the extracellular domain, is less susceptible to mutation, hence loss of reaction.

Statistical analysis

Results from the modified Banff score were statistically analyzed using MDCalc version 19.7.1 (MedCalc Software Ltd, Ostend, Belgium) using a descriptive statistical approach and compared with patient survival from symptom onset. Correlation analysis was performed using Spearman's Rho test, and Pearson's correlation coefficient survival analysis was performed utilizing the Kaplan-Meier method with 95% confidence intervals; a p-value of <0.05 was considered significant.

## Results

Demographic characteristics and duration of symptoms

A total of 40 cases fit the inclusion criteria and were selected for the study, of whom n=23 were male and n=17 were female. The mean age of the cases was 68.15 years old (median 74; mode 77, 80, and 88; range 18-91), with the mean age for males 70.30 years old (median 74; no mode; range 38-88) and mean age for females 65.24 years-old (median 63; mode 63 and 86; range 18-91). The mean duration of symptom onset to death was 14.65 days (median 14.5; mode 10; range 2-30), 15.48 days (median 15; mode 10; range 7-30) for males, and 13.53 days (median 14; mode 7; range 2-25) for females. The mean postmortem time to an autopsy was 1.63 days (median 1, mode 1, range 8 hours to 7 days).

For all patients, the gross and histomorphology spectrum of changes throughout the systems necessitated that SARS-CoV-2 be coined as the morphological culprit of tanatogenesis.

Acute renal damage indicators

**Figure 1:**
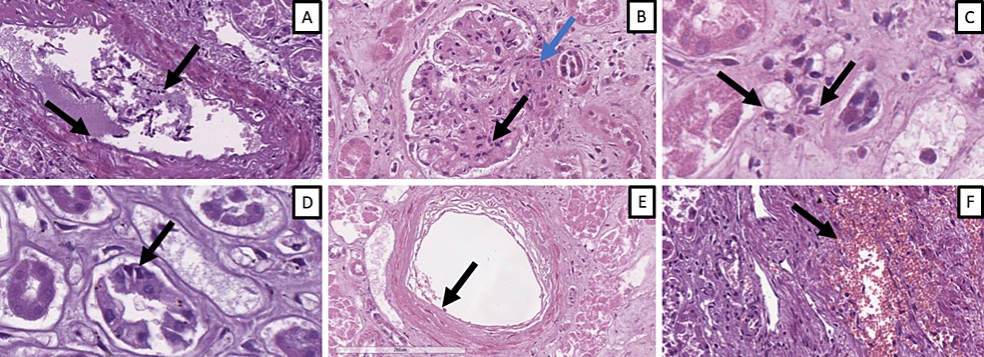
Components evaluated by the modified scoring system A: endotheliitis, edematous and desquamated in casts endothelial cells (arrows), H&E stain, original magnification x200; B: glomerulitis, inflammatory cells in the glomerular interstitium (black arrow) and mesangial matrix expansion (blue arrow), H&E stain, original magnification x400; C: capillaritis, inflammatory cells surrounding interstitial capillaries (arrows), H&E stain, original magnification x400; D: tubulitis, inflammatory cells intraepithelial inflammatory cells (arrows), H&E stain, original magnification x400; arteritis, transmural fibrinoid necrosis (arrow), H&E stain, original magnification x100; parenchymal hemorrhages, H&E stain, original magnification x200. H&E: hematoxylin and eosin

All patients presented with endotheliitis, ranging from large vessel to capillary involvement (Figure 1A). Glomerulitis was also present in all patients, with varying severity; injury score range 2-5, mean score 3.4, and mode and median 3 (affecting between 10% and 25% of sampled glomeruli) (Figure 1B). Mesangial matrix expansion was also a constant but varied in degree feature: range 2-4, mean 2.58, median 3, and mode 3 and 2 (affecting between 25% and 50% of sampled glomeruli) (Figure 1B). Cappiliaritis, although again a constant feature, was significantly more monomorphous, with a range of 2-3, mean 2.05, median, and mode 2 (between five and ten neutrophils surrounding the capillaries at most in the sampled tissues) (Figure 1 C). Tubulitis followed the same pattern as in capillaritis with a range of 2-3, mean 2.05, and median and mode 2 (between five and ten peritubular and intraepithelial inflammatory cells at most in the sampled tissues) (Figure 1 D). Arteritis, also a constant presence, was much more varied with an injury score range of 2-4, mean 3.78, median, and mode 4 (transmural fibrinoid necrosis) (Figure 1E). Interstitial fibrinoid necrosis was absent for the most part, with only three patients showing focal areas, injury score range 1-2, mean 1.08, median, and mode 1. Hemorrhages were also a significantly less common occurrence, being present in only seven patients, with an injury score range of 1-2, mean 1.18, and median and mode 1 (Figure 1F).

**Figure 2:**
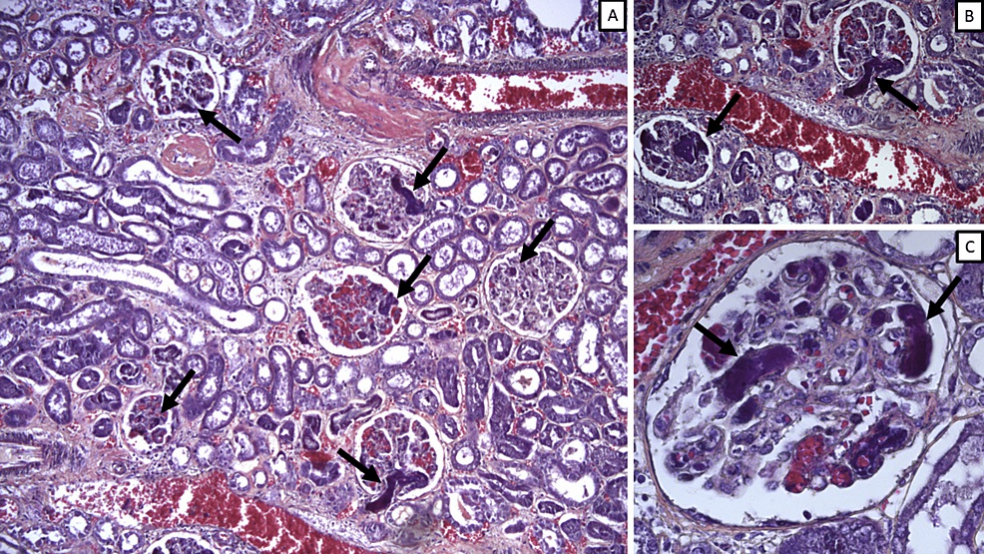
Thrombotic microangiopathy Microthrombi in glomerular capillaries (arrows), phosphotungstic acid-hematoxylin stain; A: original magnification x100; B: original magnification x200; C: original magnification x400

Thrombosis was much more varied, with an injury score range of 1-2, mean 1.33, and median and mode 1. While absent in most patients, the 13 patients (32.5%) who had thrombotic events had different forms of thrombosis: one patient (2.5%) had gross renal artery thrombosis and hence a renal infarction; eight patients (20%) had hyaline microthrombi in interstitial capillaries, and four (10%) had thrombotic microangiopathy (TMA) (Figure 2).

Chronic renal damage indicators

**Figure 3:**
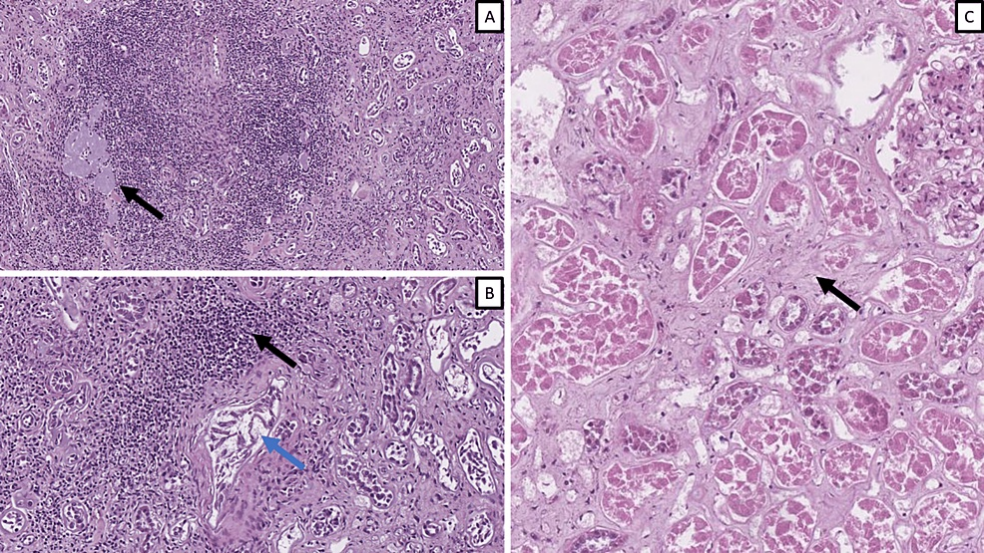
Chronic changes in renal parenchyma A: gout nephropathy, trophy and chronic inflammatory reaction (arrow), H&E stain, original magnification x50; B: interstitial nephritis (black arrow) and acute endotheliitis (blue arrow), H&E stain, original magnification x100; C: interstitial fibrosis (arrow), H&E stain, original magnification x200. H&E: hematoxylin and eosin

From the cohort, the majority of patients had signs of chronic renal damage, although the identifiable morphological cause was evident in only two, with gout nephropathy and diabetic nephropathy, while others were associated based on clinical findings with hypertension and atherosclerosis; one patient had clinically silent tuberculosis and chronic kidney disease, although no sign of tuberculosis involving the kidney was noted (Figure 3A). The indicators studies were variable, with interstitial inflammation being the most monomorphous, with an injury score range of 1-2, mean 1.2, and median and mode 1 (Figure 3B). Tubular atrophy had an injury score range of 1-4, mean 2.08, median, and mode 2. Interstitial fibrosis had an injury score range of 1-3, mean 1.83, median, and mode 2 (Figure 3C). Interstitial calcium deposits were noted in most patients with an injury score range of 1-2, mean 1.58, median, and mode 2.

Additional findings

**Figure 4:**
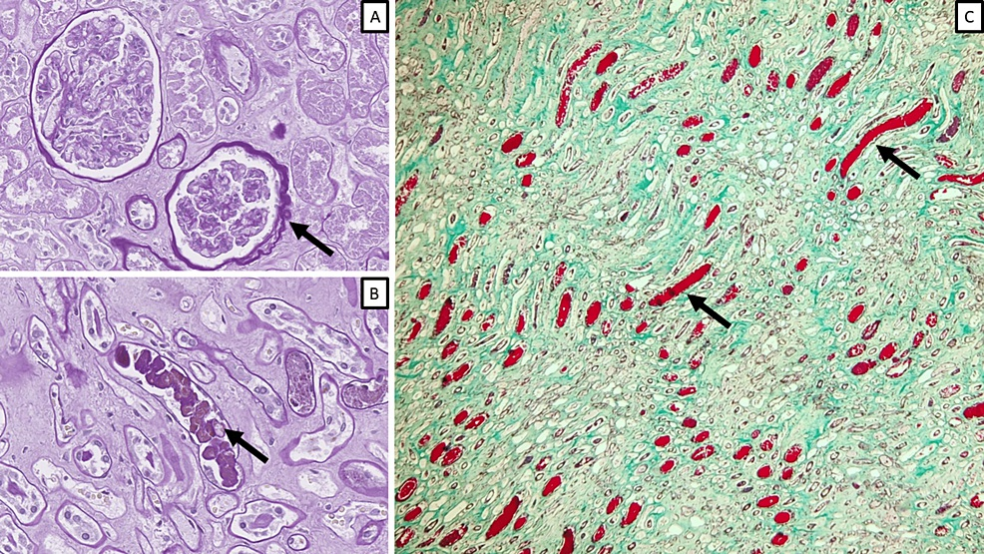
Additional morphological findings A: periglomerular fibrosis (arrow), PAS stain, original magnification x200; B: protein casts (arrow), PAS stain, original magnification x400; C: myoglobulin casts (arrows), Masson's trichrome stain, original magnification x200. PAS: periodic acid-Schiff stain

Three patients were noted to have fine periglomerular fibrosis with developing crescents, and one patient had protein casts, positive for Masson's trichrome, indicative of rhabdomyolysis (Figure 4).

Immunohistochemical analysis

**Figure 5:**
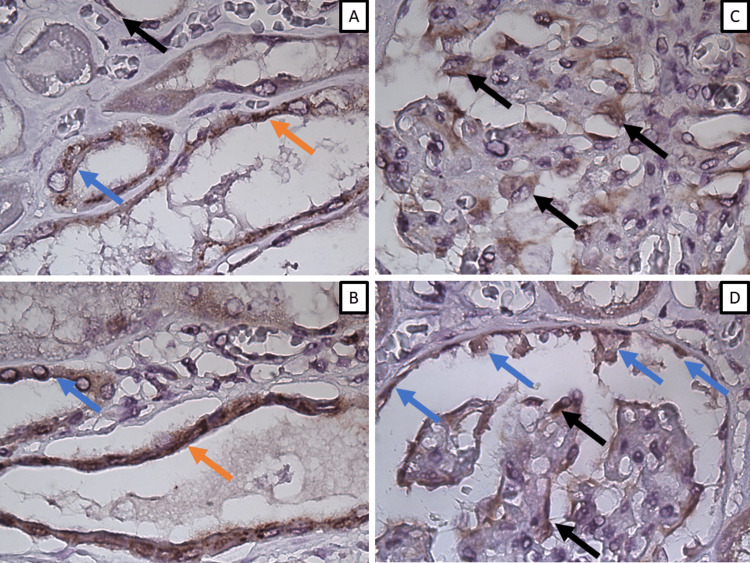
SARS-CoV-2 detection in renal structures by means of immunohistochemistry A: positivity in endothelial cells (orange arrow), proximal and distal tubular epithelial cells (blue and black arrows), original magnification x1000; B: positivity in endothelial cells (orange arrow) and proximal tubular epithelial cells (blue arrow), original magnification x1000; C: positivity in glomerular podocytes (arrows), original magnification x1000; D: positivity in podocytes (black arrows) and cells of the visceral sheet of Bowman's capsule (blue arrows).

SARS-CoV-2 spike protein was positive on immunohistochemistry in multiple renal structures (Figure 5). Unsurprisingly for the cell biology and epitheliotropism of SARS-CoV-2, there was diffuse positivity in the endothelial cells of large and small caliber blood vessels (Figure 5A-B). Further positivity was noted in the epithelial cells covering both the proximal and distant tubular system, as well as the glomerular pericytes and the parietal layer of cells of Bowman's capsule as well (Figure 5C-D)

Injury score

As already mentioned, the modified Banff scoring system used by us for the means of the study places normal kidney histopathology at 13 points, with acute indexed equaling a total of nine and chronic ones, a total of four. The maximum injury score, on the other hand, is 46, of which 31 are for acute events and 15 for chronic ones.

Across our cohort, the mean total injury score was 25.7, median and mode 26, range 22-33. The mean acute injury score was 19.03, median 19, mode 18, and range 16-22. The mean chronic injury score was 6.68, median and mode 6, and range 5-11.

Statistical analysis

**Figure 6:**
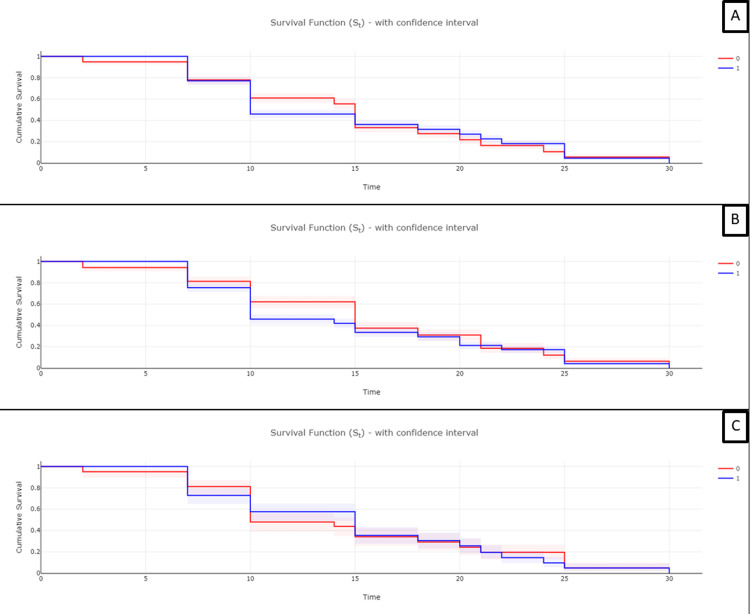
Survival analysis A: combined score analysis, no statistical significance; B: acute score, no statistical significance; C: chronic score, no statistical significance.

The overall acute and chronic injury scores were compared to the time from patient-reported symptom onset to the time of death. All statistical tests yielded no statistical significance between the type and severity of kidney injury and patient survival (p>0.05). Individual indicators were also compared to patient survival but also yielded no statistical significance. The closest values to statistical significance were between glomerulitis and arteritis, with p=0.36 and p=0.19, respectively. When the scores were divided dichotomously based on the median value, Kaplan-Meier survival analysis also showed no statistical correlation to patient survival, with the closes indicator to statistical significance being the acute injury score with p=0.245 (Figure 6).

## Discussion

The original designation of the Banff classification was implemented as a scoring tool for the rejection of organ transplants and was initially implemented in nephropathology in 1991 [2]. The modified criteria used by us for the means of the present study are based on the latest revision of the reporting system and are modified based on the aspects of the tissue sample (greater material overall compared to renal biopsy alone) and some of the known properties of SARS-CoV-2 such as its affinity toward epithelial cells in general, especially endothelial cells when discussing it outside the context of the respiratory system [2]. As the Banff criteria include both cellular and immunological phenomena of tissue damage, they combine multifaceted phenomena observed in different types of organ transplant rejection but can also be applied, as in our case for cases of virally induced damage, wherein again cellular and purely immunological phenomena can coexist and potentiate each other [1-3].

For coronaviruses as a whole, renal involvement is not a new feature, with multiple other family members also having a reported nephrotropism [3-5]. One fact to further support this is the cultivation of viral infectious agents, later determined to be coronaviruses, which were initially performed on cell cultures of human embryonal trachea and kidney [1]. Previous entries from this viral group also cause severe diseases in humans. SARS/SARS-CoV and Middle Eastern respiratory syndrome coronavirus (MERS/MERS-CoV) have also been reported to cause kidney disease. While the reposts are limited in regards to histomorphology, based on the relatively small number of patients infected and diseased from these infections and even the fewer number of autopsies performed and published, SARS is reported to cause interstitial and necrotic nephritis, with kidney involvement reportedly being a negative prognostic factor and further evidence painting toward not only the replication potential but also the tropism of this virus towards the kidney [1,6,7]. Conversely, MERS has been reported to have similar properties, with around 50% of infected cases developing into renal diseases due to acute tubular necrosis [1]. Furthermore, evidence from a patient who survived infection with MERS later developed kidney failure, with biopsy specimens from the kidney showing acute tubular necrosis and sclerosis, protein casts, and tubulointerstitial nephritis without glomerulosclerosis [8].

Significantly more evidence exists in the medical literature regarding the nephrotropism of SARS-CoV-2, based on the number of infected and diseased, the autopsies performed and published, and the social, medical, and economic sequences of the pandemic it caused, leading to unprecedented research interest, although the latter is currently dwindling [9,10]. Initially case report and small cohort either depicted no morphological changes in the kidney or attributed it to tubulointerstitial nephritis [11-13]. Later, however, based on the rapidly accumulating evidence, it became clear that there were no incidental findings but a tendency for the virus to cause a multisystem disease designated as COVID-19-associated nephropathy (COVAN) [14,15].

The depicted components of COVAN are multifaceted and regarded as related to the replication potential of the viral particles within the renal structures [14]. These are reported to be an acute tubular injury, collapsing glomerulopathy and podocytopathy, and various inflammatory changes [14,15]. Many patients are also reported with rare phenomena, such as tubular protein casts, due to rhabdomyolysis, TMA, and pauci-immune crescentic glomerulonephritis. TMA seems to be the most common rare presentation, developing in around 10% of severely ill patients [1,16].

Additional to injury in native kidneys, COVAN is also reported to affect renal transplants, with reports of antibody-mediated and acute cellular rejection, as well as acute tubular injury and TMA [16-18].

Thankfully, from a clinical point of view, most patients show steady improvement in renal function after the acute phase of COVID-19, although some of them show disease progression and require renal replacement therapy [1,18].

As confirmed by the present study results, SARS-CoV-2 has a presence in renal structures, as proven by the immunohistochemical results. Although varied in appearance to the reported focal segmental glomerulosclerosis, collapsing glomerulopathy, and interpretation acute tubular damage, the histopathological spectrum also confirms the significant histopathological alteration found in the kidney patients who died due to SARS-CoV-2 infection. However, the depictions of TMA and tubular protein casts due to rhabdomyolysis are in line with the literature data.

However, it remains in the field of scientific discussion if the reported renal transplant rejection and the findings we depicted overlap and could signify not an actual rejection but severe viral-induced damage to the more susceptible renal allograft [16-18].

As seen by our small cohort, however, the severity of histopathological alterations does not correlate with the time of survival in lethal infection, indicating that renal damage could contribute, but at least in our cohort, it does not lead to fatal outcomes within the acute infection. Clinical studies have also depicted that even in patients with impeded renal function during acute infection, there is a steady improvement in the majority of cases, while there is a small number of patients in which the clinical constellation not only persist but progresses to the need for renal replacement therapy [19-21].

Furthermore, the depicted results for viral presence and possible replication within the renal epithelia explain the presence of SARS-CoV-2 viral particles within waste wares and its correlation to the spread of the infection within communities [1,22,23].

Study limitations

A significant limitation of our study is the small number of cases included and the lack of correlation with laboratory indicators for renal function, such as creatine and urea. Furthermore, the study focuses on the morphological aspects of kidney injury without evaluating the immunological aspects to better underline the complex etiopathogenesis of viral entry, replication, and affection in the renal structures. The lack of immunological correlation is due to the retrospective nature of our study and the fact that prologued postmortem time, as seen in some patients, would make the tissues inappropriate for immunofluorescence due to postmortem lysis.

A further weak point of our study is the lack of correlation with the type of treatment received by the patients. Despite all patients reporting the initial time of symptom onset, which by itself is an imperfect tool for judging the duration of infection, the patients expired in different departments on different treatments. While some patients were intubated for a prolonged time in the respiratory ICU, some were admitted to the general ICU and intubated for a short period of time, and some expired without intubation in the respiratory, internal medicine, and infectious disease departments receiving various treatment regiments per their condition, while some expired in the emergency department shortly after presenting from home care. This heterogeneity of the cohort makes it impossible to compare the outcome and degree of damage based on the type and duration of treatment.

## Conclusions

SARS-CoV-2 as a virus has nephrotropic properties, with viral presence and possible replication within renal epithelial structures. Patients that have expired due to the infection also present with a varying severity degree of histopathological acute changes within the renal parenchyma as per the modified Banff criteria used for the study. The obtained result, however, shows no correlation between the degree and changes and the time from symptom onset to death.

As per clinical data, the depicted components of COVAN reflect significantly on kidney function. However, in most patients who survive, there is a steady, albeit sometimes incomplete, recovery of kidney function, with only a small fraction of survivors requiring renal replacement therapy.
